# Selection and validation of reference genes for quantitative real-time PCR analysis under different experimental conditions in the leafminer *Liriomyza trifolii* (Diptera: Agromyzidae)

**DOI:** 10.1371/journal.pone.0181862

**Published:** 2017-07-26

**Authors:** Ya-Wen Chang, Jing-Yun Chen, Ming-Xing Lu, Yuan Gao, Zi-Hua Tian, Wei-Rong Gong, Wei Zhu, Yu-Zhou Du

**Affiliations:** 1 School of Horticulture and Plant Protection & Institute of Applied Entomology, Yangzhou University, Yangzhou, China; 2 Laboratory for Prevention and Control of Alien Pests, Suzhou Entry-Exit Inspection and Quarantine Bureau, Suzhou, China; 3 Joint International Research Laboratory of Agriculture and Agri-Product Safety, Yangzhou University, Yangzhou, China; 4 Plant Protection and Quarantine Station of Jiangsu Province, Nanjing, China; 5 Agricultural Technology Extension Service Center of Guangling District, Yangzhou, China; Chinese Academy of Agricultural Sciences Institute of Plant Protection, CHINA

## Abstract

*Liriomyza trifolii* is a highly-invasive leafmining insect that causes significant damage to vegetables and horticultural crops worldwide. Relatively few studies have quantified gene expression in *L*. *trifolii* using real-time quantitative PCR (RT-qPCR), which is a reliable and sensitive technique for measuring gene expression. RT-qPCR requires the selection of reference genes to normalize gene expression data and control for internal differences between samples. In this study, nine housekeeping genes from *L*. *trifolii* were selected for their suitability in normalizing gene expression using geNorm, Normfinder, BestKeeper, the ΔCt method and RefFinder. *HSP21*.*7*, which encodes heat shock protein 21.7, was used as a target gene to validate the expression of candidate reference genes. Results indicated that *ACTIN* and *18S* were optimal for developmental stage and low temperature, *TUB* and *18S* showed the most stable expression for sex, and *GAPDH* and *ACTIN* were the best reference genes for monitoring gene expression at high temperature. Selection and validation of appropriate reference genes are critical steps in normalizing gene expression levels, which improve the accuracy and quality of expression data. Results of this study provide vital information on reference genes and is valuable in developing a standardized RT-qPCR protocol for functional genomics research in *L*. *trifolii*.

## Introduction

*Liriomyza* (Diptera: Agromyzidae) is a phyletic genus exhibiting replacement that continues to spread throughout the world [1–3]. *L*. *trifolii* Burgess is an invasive pest in China that has caused great losses in agricultural and horticultural crops [2–5]. In mainland China, it was initially discovered in Guangdong Province in 2005 [6–7] and has now been reported in more than ten provinces [8–10]. Both the larval and adult stages of *L*. *trifolii* damage crop plants. The larvae feed and damage the foliage, and female adults puncture plant tissue during oviposition. Both activities can reduce photosynthesis, increase defoliation, and result in yield loss [11–12].

Due to the rampant use of pesticides and onset of insecticide resistance, the development of more environmentally favorable pest management strategies is imperative. For example, strategies employing RNA interference (RNAi) or gene knockout approaches have been successfully developed for many pests [13–15]. Genetic approaches show great promise in pest management but require functional studies to identify suitable target genes and expression profiles in insects [16–19]. To successfully implement genetic strategies for control of *L*. *trifolii*, the use of standardized, real-time quantitative PCR (RT-qPCR) protocols using MIQE guidelines (Minimum Information for Publication of Quantitative Real-Time PCR Experiments) is critical [20]. RT-qPCR is widely used to analyze gene expression because of its accuracy, sensitivity, reproducibility and quantitative ability [21–22]. To accurately calculate gene expression using RT-qPCR, it is vital to use appropriate reference genes to normalize the data.

The expression of ideal reference genes should be constant in different tissues and experimental conditions. However, recent research has indicated that widely-used reference genes were differentially expressed and only stable during specific conditions [23]. *ACTIN* was one of the most stable reference genes that has been used for different developmental stages in Calliphoridae [24], but in *Frankliniella occidentalis* and *Sesamia inferens*, that gene was ranked as an unstable reference gene for certain developmental stages [17, 25]. The suitable reference genes for sex in *Coleomegilla maculate* were *16S*, *HSP70* and *RPS18*, while *18S* and *ACTIN* were relatively unstable genes [18]. For temperature treatments, *ACTIN* has been used as a stable reference gene for heat shock stress in *Drosophila melanogaster* [26], but in *Nilaparvata lugens* and *Bemisia tabaci*, *ACTIN* was ranked as one of the unstable reference genes [27–28]. Therefore, many traditional housekeeping genes are no longer valid reference genes for RT-qPCR. To our knowledge, this is the first study to evaluate the expression stability of different candidate reference genes for qRT-PCR in leaf-mining flies. Previous gene expression studies of *Liriomyza* species used *ACTIN* as the reference gene to normalize gene expression and reference genes were not selected and validated reference genes under different experimental conditions [29–32], athough the expression of *ACTIN* was monitored at different developmental stages in *L*. *sativae* [29]. It is critical, however, to validate the expression stability of reference genes under different experimental conditions before using them for normalization.

The aim of this study was to identify a suite of reference genes with stable expression in *L*. *trifolii* under different experimental conditions. Nine candidate reference genes including 18S ribosomal RNA (*18S*), β-actin (*ACTIN*), arginine kinase (*AK*), elongation factor 1α (*EF-1*), glyceraldehyde-3-phosphate dehydrogenase (*GAPDH*), histone 3 (*H3*), ribosomal protein L32 (*RPL32*), tubulin α-1 chain (*TUB*) and carbamoyl phosphate synthase (*CAD*) were evaluated for suitability in the normalization of gene expression under different experimental conditions (developmental stage, sex, and temperature). The stability and performance of the candidate reference genes was examined using geNorm [33], NormFinder [34], BestKeeper [35], the ΔCt method [36] and RefFinder [37]. The expression profile of *HSP21*.*7*, which encodes heat shock protein 21.7, was used to evaluate the suitability of different combinations of reference genes and various experimental conditions. The results provide insight regarding the selection of appropriate reference genes for functional genomic studies in *L*. *trifolii*.

## Materials and methods

### Insect cultures

Populations of *L*. *trifolii* were collected from celery (*Apium graveolens*), which was cultivated in a vegetable greenhouse located in Yangzhou (32.39°N, 119.42°E). The insect populations were maintained and reared in the laboratory for ten or more generations at 25 ± 1°C with a 16:8 h (light: dark) photoperiod as described by Chen and Kang [38]. No specific permission was required for these activities, and the field studies did not involve endangered or protected species.

### Temperature treatments

Groups of two-day-old pupae were subdivided into four repetitions (n = 30), placed in glass tubes, and exposed to heat (35°C, 37.5°C, 40°C, 42.5°C, 45°C) or cold stress (0°C, -2.5°C, -5°C, -7.5°C, -10°C) for 1 h in a constant temperature controller (DC-3010, Ningbo, China). A set of pupae maintained at 25°C was regarded as a control group. After temperature treatment, the pupae were allowed to recover at 25°C for 1 h, frozen in liquid nitrogen, and stored at -70°C.

### Developmental stage and sex

Treated stages included third-instar larvae, prepupae, two-day-old pupae, ten-day-old pupae, and adults including male and female. Each treatment (n = 30) was repeated four times.

### Candidate reference genes and primer design

Nine candidate reference genes (*ACTIN*, *AK*, *EF-1*, *H3*, *RPL32*, *18S*, *CAD*, *GAPDH*, and *TUB*) and the target gene *HSP21*.*7* were amplified from *L*. *trifolii* based on homologies with dipteran insects deposited in the NCBI database (http://www.ncbi.nlm.nih.gov). Primer Premier 5 was used for primer design using the parameters outlined by Zheng *et al*. [17]. Protocols for PCR, procedures for cloning in pGEM-T easy, and sequence analysis were conducted as previously described [39]. Sequences were submitted to GenBank, and the accession numbers are shown in Table 1.

**Table 1:** Sequence information of the candidate reference genes.

Gene name	Abbreviation	Amplicon length (bp)	Accession number
Beta-actin	*ACTIN*	322	KY231150
Arginine kinase	*AK*	426	KY563322
Elongation factor 1 alpha	*EF-1*	850	KY558636
Histone 3	*H3*	299	KY558638
Ribosomal protein L32	*RPL32*	139	KY558639
18S ribosomal RNA	*18S*	994	KY563323
Carbamoyl phosphate synthase	*CAD*	600	KY558635
Glyceraldehyde-3-phosphate dehydrogenase	*GAPDH*	996	KY558637
Tubulin alpha-1 chain	*TUB*	1350	KY558640
Heat shock protein 21.7	*HSP21*.*7*	837	KY558641

### RNA isolation

Total RNA was extracted from *L*. *trifolii* using the SV Total RNA Isolation system (Promega, WI, USA). The integrity of RNA was verified by comparing RNA bands in gels stained with ethidium bromide. Total RNA quantity and purity was determined by spectrophotometry (Eppendorf Bio Photometer plus, Hamburg, Germany).

### Expression analyses by real-time quantitative PCR

RNA (0.5 μg) was reverse-transcribed into first-strand cDNA using the Bio-Rad iScript^™^ cDNA Synthesis Kit (Bio-Rad, CA, USA). Real-time PCR reactions were performed in a 20 μl reaction volume comprised of 10 μl Bio-Rad iTaq^™^ Universal SYBR^®^ Green super mix (2×), 1 μl of each gene-specific primer (10 μM) (Table 2), 2 μl of cDNA template, and 6 μl of ddH_2_O. Reactions were carried out using a CFX-96 real-time PCR system (Bio-Rad Laboratories, Berkeley, USA) under the following conditions: 3 min at 95°C, 40 cycles of denaturation at 95°C for 30 s, and annealing at the T_m_ of primer pairs (Table 2) for 30 s. Each treatment contained four replications, and each reaction was run in triplicate.

**Table 2:** Primers used to analyze gene expression stability in *Liriomyza trifolii*.

Gene	Primers (5'to3')		Length (bp)	Efficiency (%)	R^2^	T_m_ (°C)
	Sense	Antisense				
*ACTIN*	TTGTATTGGACTCTGGTGACGG	GATAGCGTGAGGCAAAGCATAA	73	108.6	0.988	59.2
*AK*	CTTGGGTGAAGTCTACCGTCGT	GTCATCGTGAGAGAATGGCAAA	75	107.3	0.991	60.1
*EF-1*	ACTCGTCCAACTGAGAAGCCA	CACACCAGTTTCCACACGACC	98	94.5	0.998	61.0
*H3*	CCTGTAATGCCATAACTGCTGAAC	CAAAAGAGTACGGAGTTGCTGATA	117	91.09	0.998	59.5
*RPL32*	AGCACTTCATCCGCCATCAAT	ACTGACCCTTGAAACGACGAC	104	106.3	0.990	59.0
*18S*	GAAGCAGTTTGGGGGCATTA	TTGGCAAATGCTTTCGCTTA	88	100.3	0.992	55.8
*CAD*	CGATAAGTGCTATTTCTTGCCCT	GTCCACCAAAAGTCAACAAAACG	94	103.2	0.994	60.0
*GAPDH*	AGGCTGTTGGCAAAGTGATTC	CTTTTCCCAAACGCACAGTCA	110	90.2	0.999	59.1
*TUB*	TCCTTGTTGATGGAGCGATTG	GGTTGATACTTGAGGTGCGGG	86	94.9	0.994	59.6
*HSP21*.*7*	CAACAGTTTGCTCCCAATGAAG	GAGGTAGCGTCTGGAGAAGTGA	125	97.5	0.994	57.5

### Data analysis

The stability of the nine candidate reference genes was evaluated using geNorm, NormFinder, BestKeeper, the ΔCt method and RefFinder, which is a comprehensive software platform integrating all four algorithms. Pairwise variation (V), which is determined by geNorm, was used to determine the optimal number of reference genes for accurate RT-qPCR normalization. V_n_/V_n+1_ indicated the pairwise variation between two sequential normalization factors, and a cut-off threshold of V_n_/V_n+1_ = 0.15 was used for valid normalization [33]. Lower scores denote greater transcriptional stability and better suitability as a reference gene for each of the evaluated programs. RefFinder was used to select the best reference genes based on the final results of the four different programs.

### Evaluation of target gene expression

HSP21.7 is a member of the heat shock protein superfamily, which contains molecular chaperones that increase heat tolerance and protect organisms from thermal injury [29, 40]. *HSP21*.*7* was used as a target gene to evaluate the candidate reference genes. Relative expression was calculated using the 2^-ΔΔCt^ method [41]. Geometric means of the reference genes were used to normalize expression under the different experimental conditions. Statistical significance between treatments was analyzed by one-way ANOVA and further evaluated using Tukey’s multiple comparison (*P*<0.05) in SPSS v. 16.0 software (SPSS, Chicago, IL, USA).

## Results

### Amplification efficiency of candidate reference genes

For each primer pair, specific amplification was confirmed by a single peak in melting-curve analysis. A standard curve was generated for each gene and the regression correlation coefficient (R^2^) and PCR efficiency (E) for each standard curve were detailed in Table 2. All ten genes showed E values between 90.2–108.6% and R^2^ values greater than 0.988.

### Expression profile of candidate reference genes

**Fig 1:**
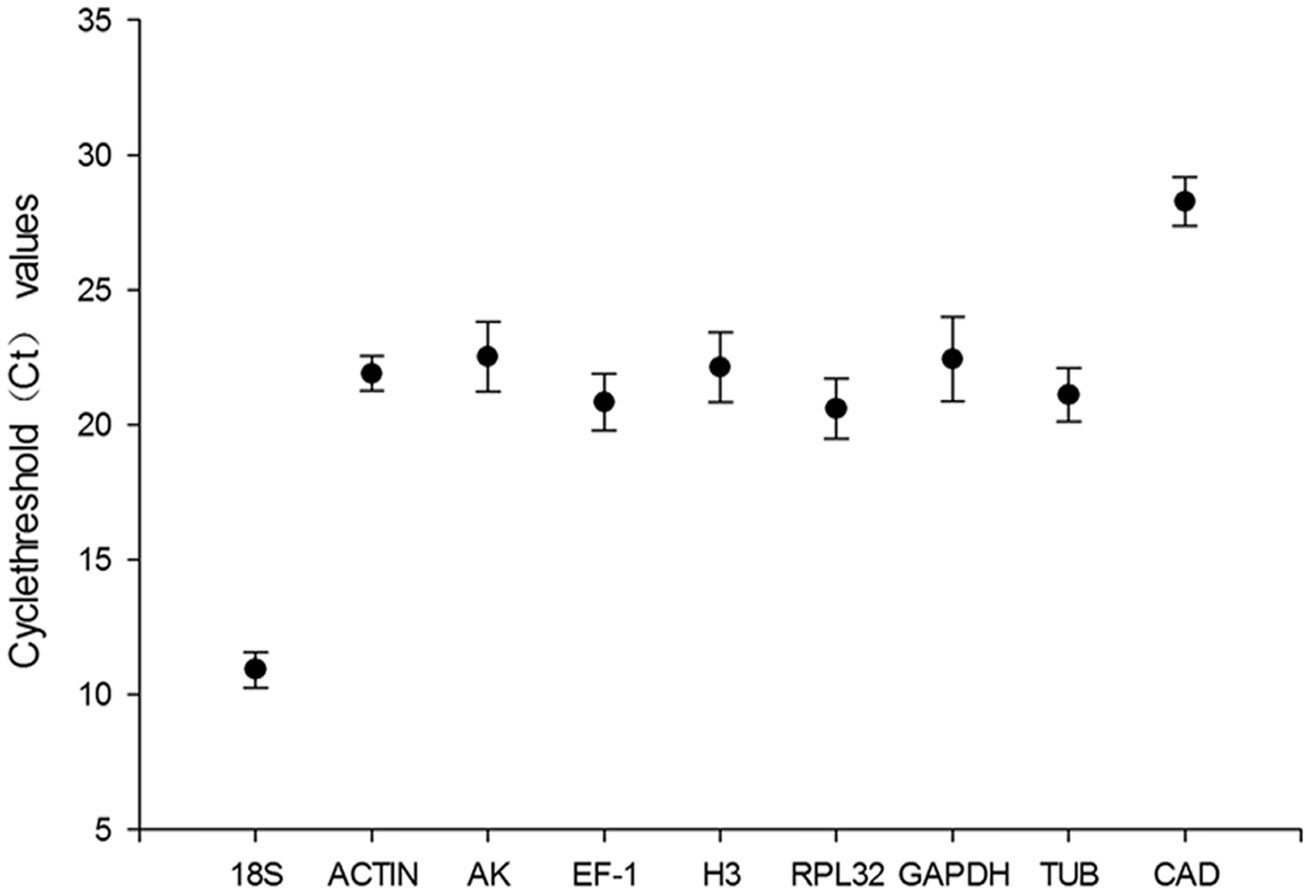
Expression profiles of the nine candidate reference genes in different samples. The black dot indicates the mean Ct value of duplicate samples, and the bars indicate the standard deviation (SD) of the mean.

The Ct values generated from the nine candidate reference genes were used to estimate the stability of gene expression across different experimental treatments. The mean Ct values of the nine reference genes varied from 10.91 to 28.27 for *18S* and *CAD*, respectively. With the exception of *18S* and *CAD*, seven of the nine candidate reference genes displayed a narrow range of mean Ct values in all experimental samples and all the standard deviation (SD) values of those reference genes were < 2.0 (Fig 1).

### Analysis of gene expression stability

**Fig 2:**
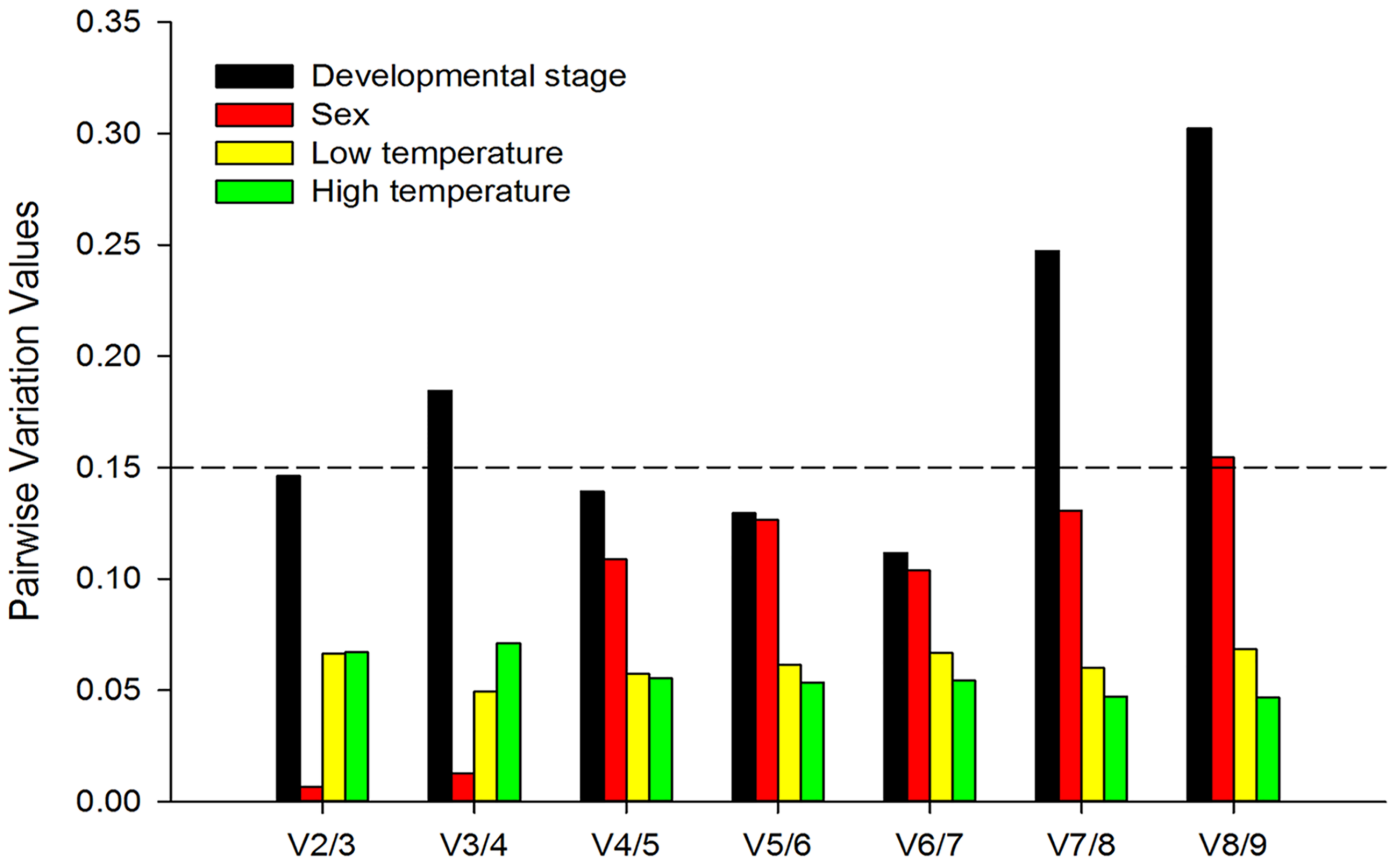
Optimal number of reference genes for normalization in *Liriomyza trifolii*. The pairwise variation (V_n_/V_n+1_) was analyzed between normalization factors NF_n_ and NF_n+1_ by geNorm program to determine the optimal number of reference genes. Values < 0.15 indicate that additional genes are not required for the normalization of gene expression.

The geNorm program uses mean expression stability values (M-values) to determine the best set of reference genes. Lower M-values indicate greater stability. geNorm ranked *ACTIN* and *18S* as the most stable genes in different insect developmental stages, and *AK* and *18S* were the most stable genes for adults of either sex (Table 3). *ACTIN* and *GAPDH* co-ranked as the most stable genes in response to low and high temperature treatments. The overall ranking of the nine reference genes is shown in Table 3. geNorm analysis revealed that the pairwise variation values were below the proposed 0.15 threshold value. The first V-value < 0.15 emerged at V2/3 (Fig 2), suggesting that two reference genes are reliable for normalization in the four experimental conditions (developmental stage, sex, low and high temperature).

**Table 3:** Ranking order of the candidate reference genes under different experimental conditions.

Experimental conditions	Rank	geNorm		NormFinder		BestKeeper		Delta Ct	
		Reference gene	Stability	Reference gene	Stability	Reference gene	Stability	Reference gene	Stability
Developmental stage	1	ACTIN/ 18S	0.3592	ACTIN	0.1245	ACTIN	0.7500	TUB	0.7771
	2			TUB	0.1535	18S	0.7916	18S	0.8308
	3	TUB	0.4349	18S	0.2324	CAD	1.0002	ACTIN	0.8809
	4	AK	0.6060	EF-1	0.4711	TUB	1.0978	EF-1	0.9161
	5	EF-1	0.6759	AK	0.6379	RPL32	1.2943	AK	0.9956
	6	RPL32	0.7419	RPL32	0.7271	EF-1	1.3081	RPL32	1.0142
	7	GAPDH	0.7923	GAPDH	0.7818	AK	1.3150	GAPDH	1.1202
	8	CAD	1.1011	CAD	1.0971	GAPDH	1.4981	CAD	1.3885
	9	H3	1.4729	H3	1.8734	H3	1.5164	H3	2.117
Sex	1	AK/ 18S	0.0048	CAD	0.0604	ACTIN	0.7710	TUB	0.4484
	2			TUB	0.1120	18S	0.7833	CAD	0.4770
	3	ACTIN	0.0148	RPL32	0.1924	AK	0.7867	GAPDH	0.4932
	4	GAPDH	0.0324	GAPDH	0.4238	GAPDH	0.8157	RPL32	0.5017
	5	TUB	0.2373	AK	0.4587	TUB	1.1742	AK	0.5041
	6	CAD	0.4111	18S	0.4628	CAD	1.4027	18S	0.5062
	7	RPL32	0.5009	ACTIN	0.4774	RPL32	1.4686	ACTIN	0.5170
	8	EF-1	0.6370	EF-1	0.5691	EF-1	1.7679	EF-1	0.6888
	9	H3	0.8045	H3	0.9544	H3	2.1046	H3	0.9833
Low temperature	1	ACTIN/ GAPDH	0.1750	18S	0.1215	18S	0.2743	ACTIN	0.2444
	2			AK	0.1518	TUB	0.3725	AK	0.2779
	3	RPL32	0.2032	ACTIN	0.1534	AK	0.4417	EF-1	0.2791
	4	AK	0.2166	EF-1	0.2027	ACTIN	0.4460	GAPDH	0.2832
	5	EF-1	0.2539	RPL32	0.2076	EF-1	0.4485	18S	0.2849
	6	18S	0.3031	GAPDH	0.2466	RPL32	0.4514	RPL32	0.3175
	7	CAD	0.3587	CAD	0.2469	CAD	0.4955	CAD	0.3401
	8	TUB	0.4025	TUB	0.2883	GAPDH	0.5509	TUB	0.3769
	9	H3	0.4595	H3	0.4212	H3	0.6086	H3	0.3819
High temperature	1	ACTIN/ GAPDH	0.0856	AK	0.0714	GAPDH	0.1843	AK	0.2216
	2			GAPDH	0.0728	ACTIN	0.1988	GAPDH	0.2328
	3	AK	0.1644	ACTIN	0.1130	AK	0.2262	ACTIN	0.2592
	4	TUB	0.2331	TUB	0.1635	RPL32	0.2411	TUB	0.2770
	5	CAD	0.2652	EF-1	0.1955	EF-1	0.2724	EF-1	0.2950
	6	H3	0.2971	RPL32	0.2324	TUB	0.2981	CAD	0.3141
	7	EF-1	0.3288	CAD	0.2383	CAD	0.3180	RPL32	0.3257
	8	RPL32	0.3549	H3	0.2610	18S	0.3406	H3	0.3442
	9	18S	0.3793	18S	0.2832	H3	0.4757	18S	0.4042

The NormFinder algorithm ranks each gene independently, and a lower stability value (SV) indicates higher expression stability. NormFinder ranked *ACTIN* and *CAD* as the most stable genes for developmental stages and sex, respectively (Table 3). *18S* was the most stable gene in response to low temperatures, whereas *AK* was best for the high temperature treatments (Table 3).

The stability of a gene is inversely proportional to the SD and coefficient variation (CV) as computed by BestKeeper. *ACTIN* was the most stable gene for developmental stages and insect sex when BestKeeper was used, whereas *18S* and *GAPDH* were the most stable genes for low and high temperature treatments, respectively (Table 3).

The ΔCt method uses raw Ct values, and the mean SD of each gene is inversely proportional to its stability. The ΔCt method indicated that *TUB* was the most stable reference gene for developmental stages and sex, whereas *ACTIN* and *AK* were the most stable genes for low and high temperatures, respectively (Table 3).

**Fig 3:**
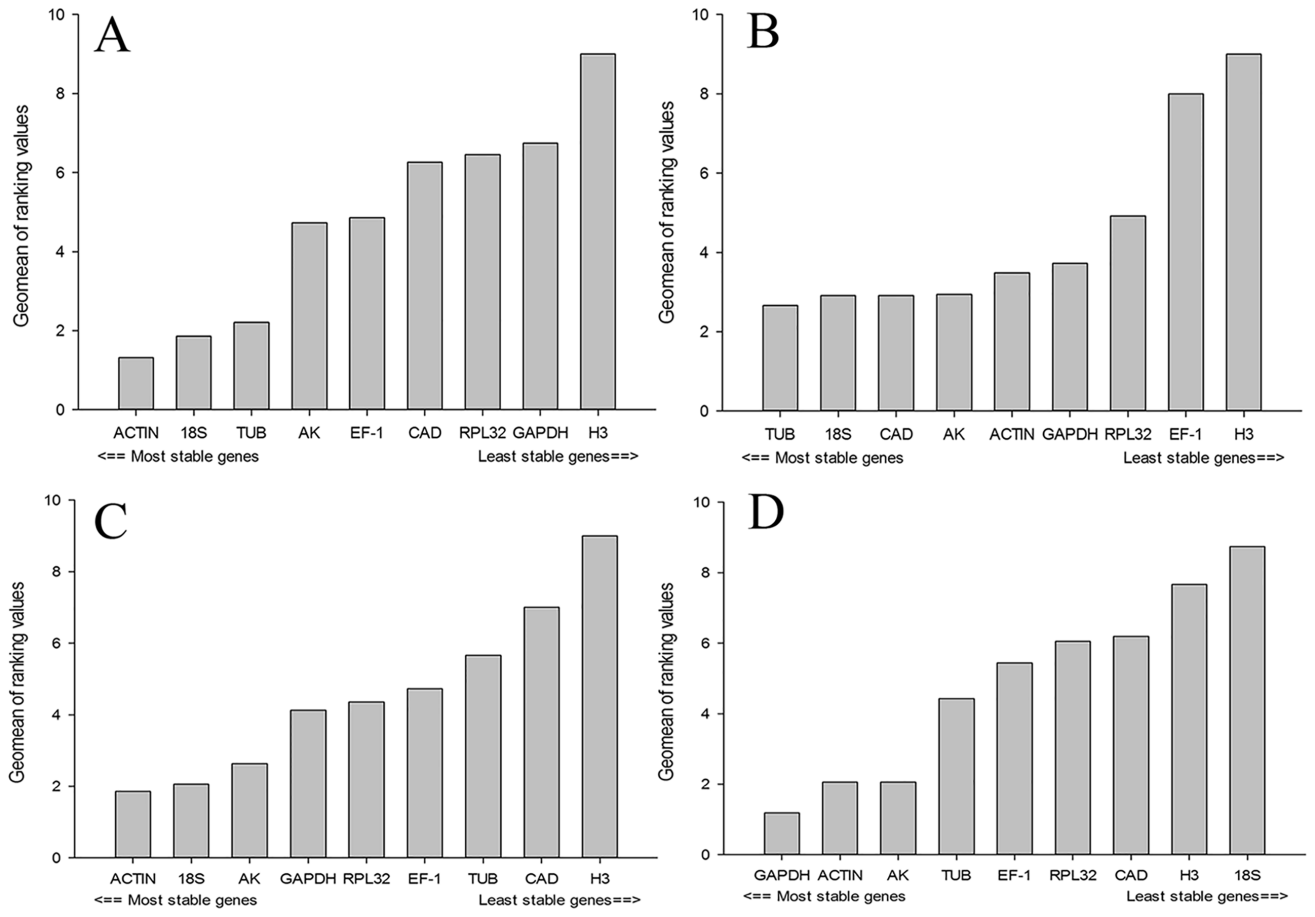
Expression stability of candidate reference genes under different treatments. A lower Geomean value indicates more stable expression according to RefFinder. (A) different developmental stages of *Liriomyza trifolii*; (B) sex for *L*. *trifolii*; (C) low temperature treatments for *L*. *trifolii*; (D) high temperature treatments for *L*. *trifolii*.

RefFinder is a comprehensive program that integrates the results obtained from geNorm, Normfinder, BestKeeper, and the ΔCt method and ranks candidate reference genes based on their stability. The following rankings are listed in order of decreasing stability. For insect developmental stages, the comprehensive ranking obtained with RefFinder was *ACTIN*, *18S*, *TUB*, *AK*, *EF-1*, *CAD*, *RPL32*, *GAPDH*, and *H3* (Fig 3A). The stability ranking for insect sex was *TUB*, *18S*, *CAD*, *AK*, *ACTIN*, *GAPDH*, *RPL32*, *EF-1*, and *H3* (Fig 3B). The stability ranking in *L*. *trifolii* exposed to low temperatures was *ACTIN*, *18S*, *AK*, *GAPDH*, *RPL32*, *EF-1*, *TUB*, *CAD*, and *H3* (Fig 3C), whereas the ranking for high temperatures was *GAPDH*, *ACTIN*, *AK*, *TUB*, *EF-1*, *RPL32*, *CAD*, *H3*, and *18S* (Fig 3D). The ideal reference genes in response to different experimental conditions are as demonstrated by RefFinder all showed in Table 4. The optimal number of reference genes was based on geNorm.

**Table 4:** Recommended reference genes for various experimental conditions.

Experimental conditions*	Optimal number of reference genes	Recommended Reference Genes
Developmental stage	2	*ACTIN*, *18S*
(L, PP, P, OP, M, FM)		
Sex	2	*TUB*, *18S*
(M, FM)		
Low temperature	2	*ACTIN*, *18S*
(0°C, -2.5°C, -5°C, -7.5°C, -10°C)		
High temperature	2	*GAPDH*, *ACTIN*
(35°C, 37.5°C, 40°C, 42.5°C, 45°C)		

* L: third-instar larvae; PP: prepupae; P: two-day-old pupae; OP: ten-day-old pupae; M: male adult; FM: female adult.

### Validation of reference gene selection

**Fig 4:**
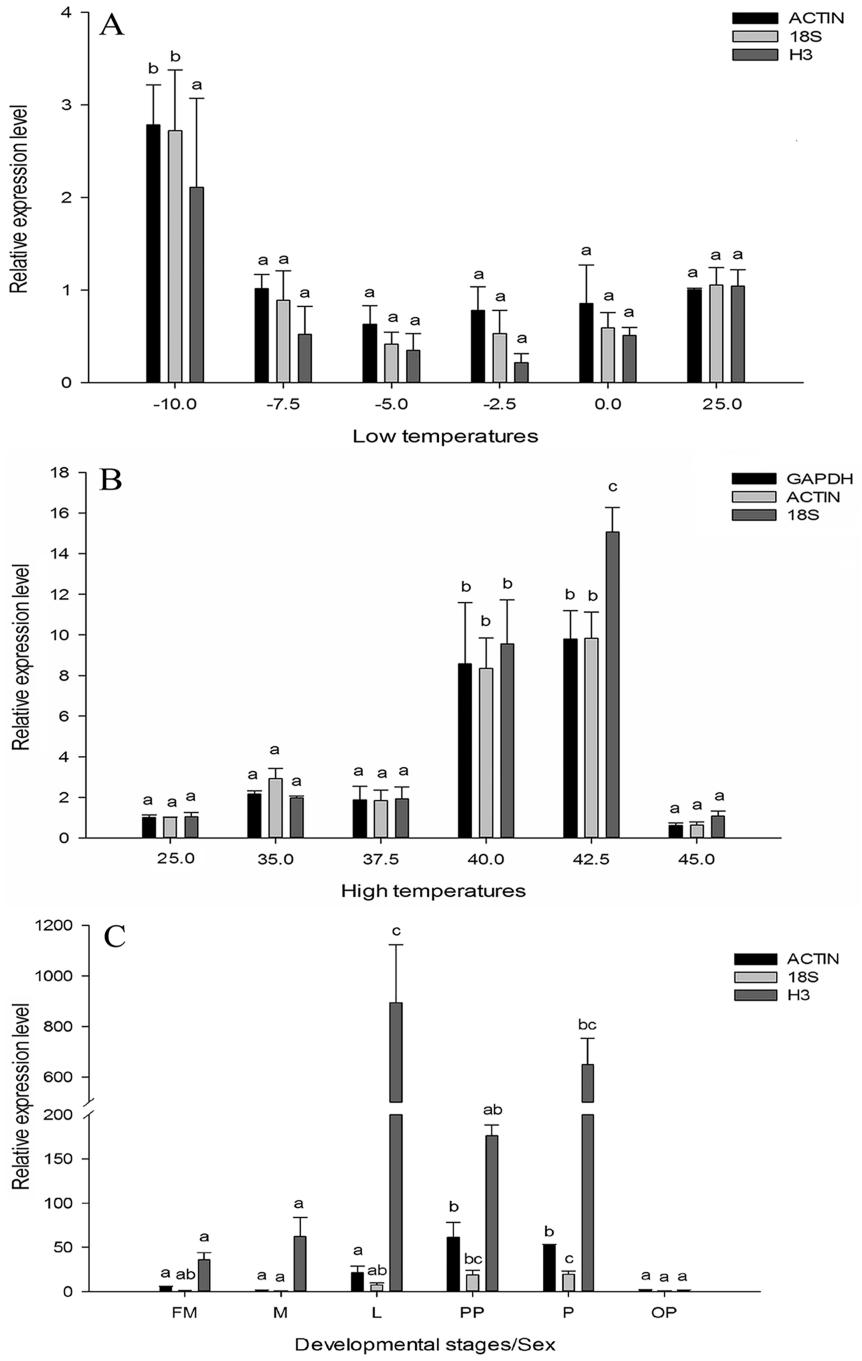
Validation of reference gene selection. (A) Relative expression levels of *HSP21*.*7* in low temperature treatments; (B) Relative expression levels of *HSP21*.*7* in high temperature treatments; (C) Relative expression levels of *HSP21*.*7* in developmental stage/sex treatments. Abbreviations: FM: female adult; M: male adult; L: third-instar larvae; PP: prepupae; P: two-day-old pupae; OP: ten-day-old pupae.

The relative expression of *HSP21*.*7* was used to assess the validity of selected reference genes. The expression level of *HSP21*.*7* was compared using the two most stable reference genes (*ACTIN* and *18S*) and the most unstable gene (*H3*), which is generally recommended for normalization by RefFinder. When *H3* was used to normalize *hsp21*.*7* expression levels in *L*. *trifolii* exposed to low temperatures, no significant differences were observed among the different temperatures (*F*_5,18_ = 2.714, *P* = 0.054). However, when the two most stable reference genes, *ACTIN* and *18S*, were used to normalize the data, relative expression was significantly higher at -10°C (*F*_5,18_ = 7.892, *P*<0.05; *F*_5,18_ = 6.609, *P*<0.05) (Fig 4A). When *HSP21*.*7* expression was evaluated in response to high temperatures, expression at 40°C and 42.5°C was significantly higher than it was at the other temperatures, regardless of the gene used for normalization (Fig 4B). Interestingly, when *hsp21*.*7* expression was normalized using the least stable gene (*18S*), the relative expression at 40°C and 42.5°C was significantly different (*F*_5,18_ = 31.399, *P*<0.05). However, expression at these two temperatures was not significantly different when the most stable genes, *GAPDH* and *ACTIN*, were used to normalize the data (*F*_5,18_ = 8.633, *P*<0.05; *F*_5,18_ = 21.489, *P*<0.05).

The relative expression of *hsp21*.*7* was also estimated for different insect developmental stages and sex, and gene expression was normalized using *ACTIN* and *18S* (the two most stable genes) and *H3* (the most unstable gene). When *ACTIN* and *18S* were used for normalization, expression of *hsp21*.*7* was highest at the prepupae and two-day-old pupae stages (*F*_5,17_ = 12.568, *P*<0.05; *F*_5,17_ = 18.381, *P*<0.05) and significant differences were observed between the other developmental stages and sexes (Fig 4C). However, *hsp21*.*7* expression was highest at the third-instar larval stage when *H3* was used as a reference gene (*F*_5,17_ = 12.572, *P*<0.05), and there were huge differences in *hsp21*.*7* expression using the stable and unstable reference genes (Fig 4C).

## Discussion

Biological samples often show great differences in the quality of RNA and the efficiency of reverse transcription. Consequently, the selection of appropriate reference genes is imperative in reducing error [42]. It is now apparent that a stable, constant level of expression does not exist for housekeeping genes in different insect species or within the same species subjected to different experimental conditions. Although RT-qPCR is widely used in gene expression studies because of its speed, accuracy and sensitivity [43–44], our results demonstrate that the correct choice of stable reference genes is required to ensure the reliability of the results.

Several studies on reference gene validation have emphasized that multiple internal genes must be evaluated to improve the accuracy of qRT-PCR analysis and the interpretation of gene expression [28, 45–46]. In this study, nine genes were analyzed for their suitability as reference genes for qRT-PCR in *L*. *trifolii* that differed in developmental stage, sex and temperature stress. The computational programs geNorm, NormFinder, BestKeeper and the ΔCt method were used to generate stability rankings for the nine genes. These varied slightly between the four methods (Table 3). It is worth mentioning that, in addition to ranking function, the geNorm also has the function of selected optimal number of reference genes [33]. More and more researchers have realized that only a single reference gene with high expression stability may be not enough for normalization of gene expression under some experimental conditions, so in most experimental conditions two or more reference genes were required for accurate and reliable results. The comprehensive tool RefFinder was utilized to evaluate the rankings of the four programs and to generate a final ranking for the different experimental conditions (Fig 3).

RefFinder analysis indicated that *ACTIN* and *18S* were the most stable reference genes for *L*. *trifolii* at different developmental stages, whereas *TUB* and *18S* were the most stable genes when comparing male and female adults. *GAPDH* and *ACTIN* were optimal reference genes at high temperatures, whereas *ACTIN* and *18S* exhibited the most stable expression at low temperatures. Consistent with the reference genes validated for other insect organisms [26–27, 47–49], our study recommended *ACTIN*, *18S*, *TUB* and *GAPDH* were the better genes for normalizing expression in *L*. *trifolii* exposed to different experimental treatments. It is noteworthy that previous reference gene validation studies combined high and low temperature treatments, collectively referring to them as temperature treatment [26–27, 50]. Interestingly, we discovered that the reference gene stability rankings for high and low temperatures were different and often contrasted. In this study, *18S* was ranked as a stable reference gene for low temperature, but was the least stable reference gene for high temperatures. These results are likely relevant in the expression of *HSPs* in leafminer species. For example, *hsp60* in *Liriomyza* spp. responded to cold but not heat stress [30]. Consequently, the use of reference genes selected from combined heat/cold temperature stress could cause inaccuracies in the normalization of *HSP60* expression. Thus, the stability of reference gene expression in *L*. *trifolii* needs to be carefully examined for experimental conditions and should be considered in the context of insect biology and physiology whenever possible.

Heat shock proteins (HSPs) play important roles in the environmental adaptation of various organisms. *HSP21*.*7* is a member of the small HSPs family. It along with a number of other small HSPs play a role in temperature tolerance and development in insects [29, 40]. For example, *HSP21*.*7* was significantly induced by temperature stress and developmental processes in *L*. *sativae* [29]. In our study, the expression of *hsp21*.*7* was investigated to validate the ranking of reference genes by RefFinder. When the two most stable genes were used for normalization under different experimental treatments, the *HSP21*.*7* expression pattern was consistent with *L*. *sativae*. However, when the least stable gene was used, the normalized expression was significantly different. Therefore, the selection of optimal reference genes for normalization is critical, especially when differences in expression levels are subtle. In order to generate reliable expression results with a given target gene, the reference gene(s) must be stably expressed under the specific experimental conditions. If the appropriate reference genes are not selected, expression of the target gene may be biased and show significant differences that lead to incorrect conclusions [51–52].

The emerging availability of genomic sequencing, gene chips, and databases provide new approaches to more accurately select reference genes [53–55]. Former housekeeping genes with unstable expression will be gradually replaced by new reference genes that have been evaluated under experimental conditions. This study validated several reference genes for *L*. *trifolii* and provides an approach that should be considered when screening reference genes in other species.
